# Detecting imported malaria infections in endemic settings using molecular surveillance: current state and challenges

**DOI:** 10.3389/fepid.2025.1490141

**Published:** 2025-02-26

**Authors:** Mahdi Safarpour, Luis Cabrera-Sosa, Dionicia Gamboa, Jean-Pierre Van geertruyden, Christopher Delgado-Ratto

**Affiliations:** ^1^ Malaria Research Group (MaRch), Family Medicine and Population Health Department, Faculty of Medicine and Health Sciences, Global Health Institute, University of Antwerp, Antwerp, Belgium; ^2^ Laboratorio de Malaria: Parásitos y Vectores, Laboratorios de Investigación y Desarrollo, Facultad de Ciencias e Ingeniería, Universidad Peruana Cayetano Heredia, Lima, Peru; ^3^ Grupo Malaria: Epidemiología Molecular, Instituto de Medicina Tropical “Alexander von Humboldt”, Universidad Peruana Cayetano Heredia, Lima, Peru

**Keywords:** malaria elimination, metapopulation, population genetics, travel-acquired malaria, genomic surveillance

## Abstract

The Global Technical Strategy for Malaria 2016–2030 targets eliminating malaria from at least 35 countries and reducing case incidence by 90% globally. The importation of parasites due to human mobilization poses a significant obstacle to achieve malaria elimination as it can undermine the effectiveness of local interventions. Gaining a comprehensive understanding of parasite importation is essential to support control efforts and advance progress toward elimination. Parasite genetic data is widely used to investigate the spatial and temporal dynamics of imported infections. In this context, this systematic review aimed to aggregate evidence on the application of parasite genetic data for mapping imported malaria and the analytical methods used to analyze it. We discuss the advantages and limitations of the genetic approaches employed and propose a suitable type of genetic data along with an analytical framework to discriminate imported malaria infections from local infections. The findings offer potential actionable insights for national control programs, enabling them select the most effective methods for detecting imported cases. This also may aid in the evaluation and refinement of elimination programs by identifying high-risk areas and enabling the targeted allocation of resources to these regions.

## 1 Introduction

Over the last decade, the technical framework for malaria control and elimination recommended by the World Health Organization (WHO) has substantially reduced the global malaria burden (1). Despite all efforts, the number of endemic countries with fewer than 10,000 malaria cases per year increased from 26 in 2000 to 47 in 2020 (2). Likewise, malaria cases increased from 227 million in 2019 to 249 million in 2022 (3). The relative increase in the number of malaria cases is favored by malaria parasite importation due to human mobility from one malaria endemic region to the other (4). From a fundamental perspective, this scenario reflects the metapopulation dynamics theory, where parasite populations are divided into geographically separate groups across different regions but with limited interaction between them. In the context of metapopulation dynamics, human mobility facilitates parasite importation and recolonization in areas where the parasite population was (nearly) extinct due to successful interventions (5). Therefore, it is essential to quantify the role of human mobility in the spatial distribution and connectivity of malaria parasites in countries aiming for malaria elimination (6).

To unravel how human mobility impacts the geographical spread of malaria, researchers often collect data on recent travel history in clinical cases (7). In theory, this this approach helps identify regions from which the disease is being imported and enables the design of targeted interventions. However, the reliability of travel survey data depends on the respondents’ ability to accurately recall their travel history (8). Therefore, recall bias in travel survey data can limit their application to correctly determining the origin of malaria infection. In contrast, mobile phone data and Global Positioning System (GPS) tracking can provide reliable data to measure the spatial spread of malaria parasites (9). Nevertheless, the application of mobile phone data is limited to the locations where cell towers exist (10). In addition, GPS tracking requires devices comfortable enough to carry for long periods, replacing or recharging batteries quite frequently, and relies upon receiving high-quality signals from satellites, among other logistics, which limits its usability.

Current efforts involve the use of parasite genetic data to determine the origin of malaria infections. In principle, the genetic information of parasites can potentially provide the most direct measure of parasite connectivity and can lead to estimating the sources and sinks of malaria parasites (11). Various molecular techniques are available to analyze and profile the genetic data of malaria parasites. Microsatellite genotyping is one of the most widely used molecular methods to study the genetic diversity and population structure of malaria parasites, particularly in regions such as the Peruvian Amazon and West Africa (12, 13). Other methods, such as single nucleotide polymorphisms (SNPs) profiling, are widely used to explore parasite population structure in regions like Papua New Guinea (14) and at the Myanmar-China border (15). In recent years, advancements in sequencing technologies have established whole-genome sequencing (WGS) as a powerful tool for investigating malaria transmission dynamics. This approach has been applied not only at the continental scale but also at regional levels with smaller spatial scales, such as those in Southeast Asia (16).

The findings of these studies highlight the potential of genetic markers to provide valuable insights into how human migration may influence the spread of malaria infection. One of the earliest studies in this context, conducted by Griffing et al., demonstrated that Peruvian *P. falciparum* populations may have expanded from bottlenecked populations or migrants during the post-eradication era (17). Similarly, Vera-Arias et al. revealed that the limited number of *P. falciparum* clonal types circulating in northwest Ecuador are related to ancestral parasite clonal lineages reported in the Pacific Coast. However, the study did not conclusively determine whether the source of infection was due to human migration from neighboring regions or residual clonal types circulating in the country in low proportions (18).

Despite the widespread use of various molecular techniques in malaria research, there remains limited information on their effectiveness in accurately identifying imported cases and assessing the role of human migration in sustaining malaria transmission. This gap is particularly noted at smaller geographical scales, such as within countries, where parasites are expected to be closely related. Consequently, this systematic review provides an overview of the current evidence, focusing on the types of genetic data used to study imported malaria and the methods used for their analysis. It also highlights the strengths and limitations of each approach and outlines a roadmap for future research. The findings can provide comprehensive evidence for epidemiologists and other health scientists on the reliability of malaria parasite genetic data to map the imported individuals and provide actionable insights for policymakers to eliminate malaria.

## 2 Materials and methods

### 2.1 Search strategy

Four online bibliographic databases were searched: PubMed, Scopus, Cochrane Library, and Web of Science. We developed a comprehensive search strategy based on the keywords related to malaria parasite species, human mobility, and molecular/genomic tools used to map the spatial spread of malaria infection. This review was restricted to studies reported in English, without considering any time limitation, and included literature up to the end of November 2024. Full details of the search strategy are available in Supplementary Appendix 1. Furthermore, reference lists of included articles were screened to identify relevant studies that were not found through our initial search.

### 2.2 Study selection

**Figure 1:**
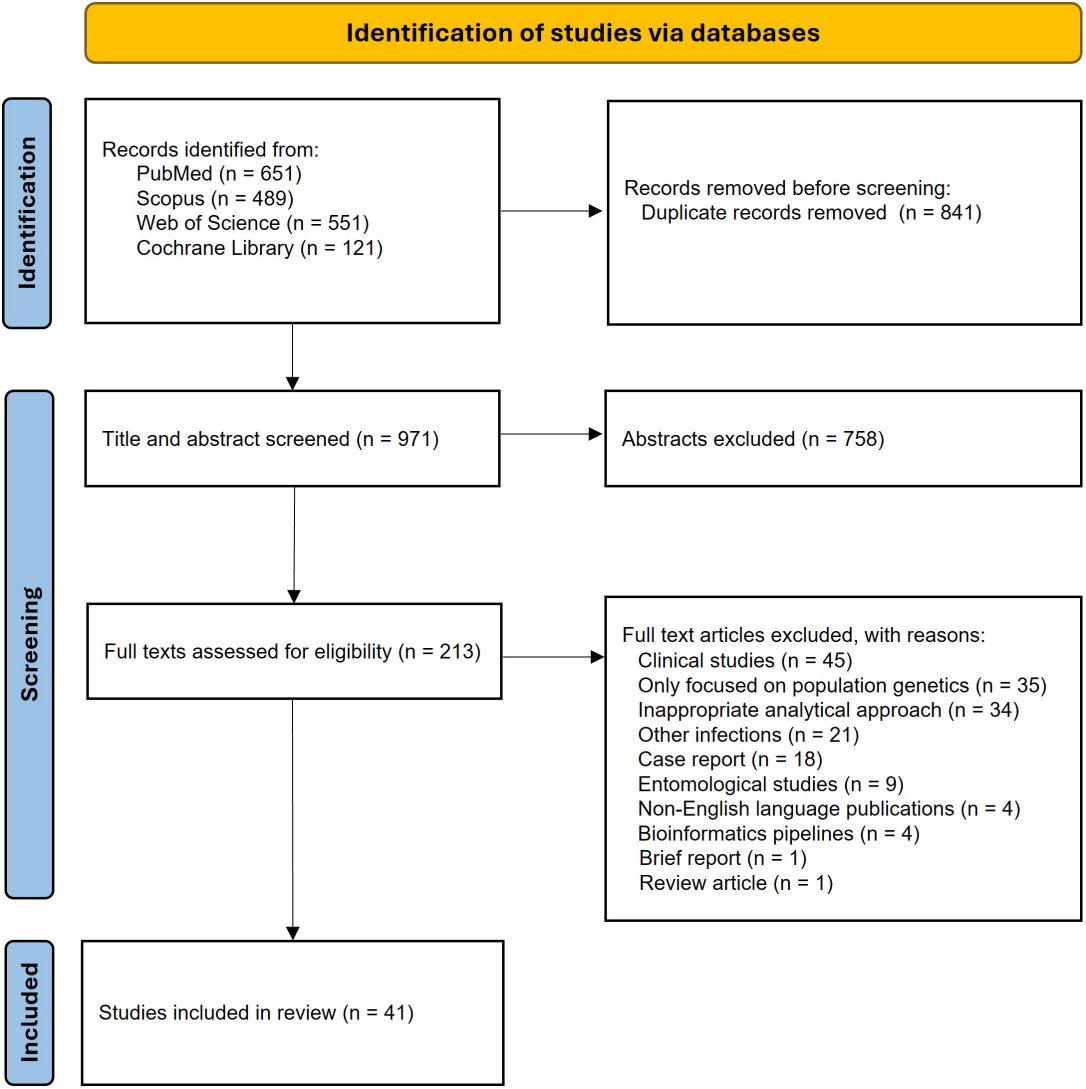
PRISMA flow diagram showing the steps followed to select eligible articles, along with the number of articles retained at each stage.

Articles from online bibliographic databases were stored and combined as EndNote files, where duplicate articles were removed. The titles and abstracts of articles were screened. Abstracts were excluded if they focused exclusively on the clinical aspects of malaria infections, addressed infections other than malaria, were written in languages other than English, or were not relevant to genomic surveillance of malaria. Articles selected for full-text review were independently assessed by two reviewers for final inclusion, and any disagreements were resolved through discussion and consensus. Studies were included if they were peer-reviewed articles, used parasite genetic data, focused on either *Plasmodium vivax* or *Plasmodium falciparum*, and included an analytical framework that investigated the origin of infection or explicitly analyzed connections between geographical areas, either at a global or regional scale. Articles were excluded if they only descriptively reported the genetic marker without integrating them into an analytical framework, or if they addressed unrelated topics, as detailed in the PRISMA flowchart (Figure 1).

### 2.3 Data extraction

Data extraction was done from the full text of all eligible articles. Data related to study setting, species of malaria parasites, sample size, and type of genetic markers were extracted. Furthermore, the available data on drug resistance markers, travel surveys, and GPS tracking were recorded for each study. The analytical methods used for analyzing the parasite genetic data were also collected.

### 2.4 Data synthesis

Data were synthesized narratively, and descriptive statistics were used to summarize the main study characteristics, such as study setting, sample size, and the number of genetic loci examined. Studies were grouped according to the following three criteria: (1) species of malaria parasites (*Plasmodium vivax* or *Plasmodium falciparum*); (2) type of genetic marker (Microsatellite, Single Nucleotide Polymorphism, Whole-Genome Sequencing, or Targeted Sequencing) and (3) analytical approach (genetic diversity, population structure, and relatedness among populations or individuals). The findings were presented and discussed descriptively, categorizing them based on the type of genetic marker used for genotyping and the analytical approach employed to determine the origin of infection. Studies were then categorized by geographical region (continent) and publication timeline. This approach was selected to offer a clear overview of how methods have evolved over time in specific regions, highlighting the number of genetic markers used and changes in study scope.

## 3 Results

A total of 1,812 articles were initially retrieved from the databases, resulting in 971 unique publications after removing duplicates. We first screened the titles and abstracts, and 758 articles were excluded and did not proceed to the full-text review. We identified one additional article by manually searching the reference lists of included articles. Out of 213 studies selected for full-text screening, 172 studies were excluded because they did not meet the inclusion criteria. The full-text review resulted in the identification of 41 eligible studies. The PRISMA flow chart illustrating the screening process is summarized in Figure 1.

### 3.1 General characteristics of included studies

**Figure 2:**
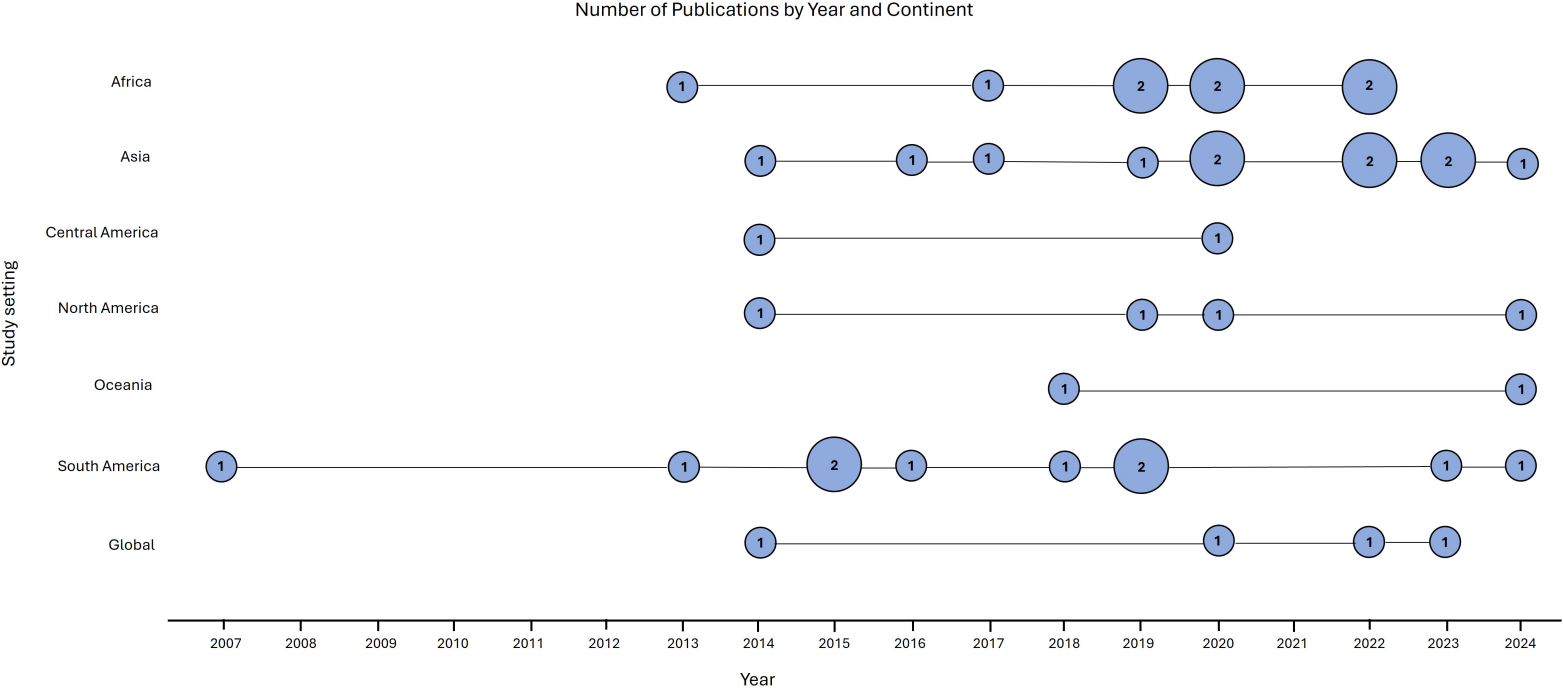
The timeline of eligible studies published across each continent over time.

The eligible articles for this review were published between 2007 and 2024. Only one study was published between 2007 and 2012 (19). However, from 2013 to 2024, there was an increase in the number of publications across all continents (Figure 2). This indicated a rising interest in applying genetic methods to distinguish between indigenous and imported cases, which is particularly important for countries approaching zero malaria cases (20). The highest number of publications were from Asia and South America, each with ten, followed by Africa with nine (Table 1). In contrast, Central America and Oceania had the fewest publications, with just two each. The sample size varied between 45 (21) to 8,654 subjects (22). There were 19 studies focused on *P. falciparum* and 18 on *P. vivax*. Additionally, four studies investigated both *P. falciparum* and *P. vivax* concurrently. Out of 41 studies, the majority (*n* = 22) focused on a national scale, using genetic data to finely map imported malaria cases within their respective countries.

**Table 1:** Overview of the eligible studies selected for this review article.

Author	Year of publication	Country	Continent*	Study scale	Parasite	Sample size	Genotyping*	Drug resistance marker	Travel survey	Mobile data
Osorio	2007	Colombia	South America	Within country	Pf	679	MS (5)		Yes	
Khaireh	2013	Djibouti	Africa	Within country	Pf	181	MS (5)+SNPs (5)	Yes		
Griffing	2013	Brazil	South America	Within country	Pf	190	Targeted sequencing	Yes		
Preston	2014			Global	Pf	711	SNP (23)		Yes	
Rodrigues	2014	USA	North America	Global	Pv	417	WGS		Yes	
Liu Yaobao	2014	China	Asia	Within country	Pv	128	MS (7)		Yes	
Patel	2014	Guatemala	Central America	Global	Pf	86	MS (8)	Yes	Yes	
Baldeviano	2015	Peru	South America	Within country	Pf	54	MS (7)	Yes	Yes	
Chenet	2015	Colombia	South America	Within country	Pf	257	MS (12)	Yes		
Delgado-Ratto	2016	Peru	South America	Within country	Pv	292	MS (14)			
Hamedi	2016	Iran	Asia	Cross-border	Pv	100	Targeted sequencing	Yes	Yes	
Lo	2017	China	Asia	Cross-border	Pv	895	MS (11)		Yes	
Lo	2017	Ethiopia	Africa	Within country	Pv + Pf	431	MS (14)	Yes		
Ventocilla	2018	Peru	South America	Cross-border	Pv	145	MS (5)			
Fola	2018	Papua New Guinea	Oceania	Within country	Pv	219	MS (10)			
Chang	2019	Bangladesh	Asia	Within country	Pv + Pf	1412	SNPs (101)	Yes	Yes	Yes
Roh	2019	Kingdom of Eswatini	Africa	Within country	Pf	582	MS (26)		Yes	
Pacheco	2019	Colombia	South America	Within country	Pv	624	MS (8)			
Manrique	2019	Peru	South America	Within country	Pv	777	MS (16)			
Schmedes	2019	USA	North America	Global	Pf	265	WGS		Yes	
Tessema	2019	Namibia	Africa	Cross-border	Pf	4643	MS (26)		Yes	Yes
Dewasurendra	2020	Sri Lanka	Asia	Within country	Pv	51	SNPs (40)		Yes	
Liu Yaobao	2020	China	Asia	Global	Pf	602	MS (26)		Yes	
Valdivia	2020	Honduras	Central America	Global	Pv + Pf	48	SNP (137 + 117)	Yes	Yes	
Buyon	2020	Panama	North America	Within country	Pv	59	WGS		Yes	
Ba	2020	Mauritania	Africa	Global	Pf	100	SNP (38)	Yes	Yes	
Morgan	2020	Tanzania	Africa	Cross-border	Pf	125	WGS	Yes	Yes	
Benavente	2020			Global	Pv	433	WGS		Yes	
Kattenberg	2022	Vietnam	Asia	Global	Pv	402	WGS	Yes	Yes	
Markwalter	2022	Kenya	Africa	Within country	Pf	2521	Targeted sequencing		Yes	
Atuh	2022	Cameroon	Africa	Within country	Pf	232	MS (9)		Yes	
Trimarsanto	2022			Global	Pv	799	SNPs (33-50-55)			
Cui	2022	China	Asia	Cross-border	Pv	152	Targeted sequencing			
Li	2023	China	Asia	Global	Pv	45	WGS		Yes	
Phelan	2023			Global	Pv + Pf	8654	SNP (274)	Yes	Yes	
Xu	2023	China	Asia	Global	Pf	403	Targetedsequencing		Yes	
Ruybal-Pesantez	2023	Ecuador	South America	Cross-border	Pf	58	Targeted sequencing		Yes	
Holzschuh	2024	Ethiopia	Africa	Within country	Pf	187	Targeted sequencing	Yes	Yes	
Kattenberg	2024	Peru	South America	Within country	Pv	230	SNP (33)	Yes		
Sekine	2024	Vanuatu	Oceania	Within country	Pv	69	MS (5)			
Pierre-Louis	2024	USA	North America	Global	Pf	234	Targeted sequencing		Yes	

* The numbers in parentheses represent the total number of genetic markers used.

We identified seven studies focused on cross-border investigations. Only four studies conducted global-level analyses, collecting data from around the world. The majority of studies (*n* = 17, 41%) used microsatellite markers. Eight studies used SNP markers/barcodes, and only one combined the data of both microsatellite and SNP markers (23). Seven studies employed whole genome sequencing (WGS): three focused on sequencing the entire *Plasmodium* mitochondrial genome, while others sequenced the genome of *P. vivax* and *P. falciparum*. In addition, we identified eight studies that employed targeted genome sequencing primarily focusing on drug resistance genes (*n* = 4) and genes involved in the parasite's life cycle and immune evasion (*n* = 4). Fifteen studies included drug resistance markers in their analysis (Table 1).

More than half of the studies (*n* = 28, 68%) collected travel survey data using a self-reported questionnaire. Only two studies collected mobile phone data (4, 24). The remaining studies did not explicitly mention the collection of travel survey data, mobile data, or GPS tracking data. Regarding the analytical methods, expected heterozygosity (HE) was the most commonly used to assess genetic diversity, applied in 18 studies (44%). The Admixture model was utilized in 16 studies (39%) to analyze population structure, while Principal Component Analysis (PCA) was employed in 13 studies (32%). Machine learning approaches, such as Naive Bayes and Random Forests, were used in four studies to predict the geographic origin of infections. Finally, only one study applied mathematical modeling to assess whether parasite importation could sustain malaria transmission.

### 3.2 Microsatellite markers

Seventeen studies worldwide have investigated the efficiency of MS to determine the genetic diversity and transmission dynamics of malaria parasites. Among these studies, seven were conducted in South America: four in Peru and three in Colombia. The primary focus of studies conducted in Peru was the Amazon region. The earliest study in this region was conducted in 2015 by Baldeviano et al. They genotyped seven microsatellite loci in 54 *P. falciparum* isolates to identify the source of the outbreak occurred in Tumbes, on northern coast of Peru. The genotype of the parasites from Tumbes was identical to that of all 11 *P. falciparum* isolates collected from Loreto. The findings suggested that the outbreak in Tumbes may have originated in Loreto (25). In 2016, Delgado-Ratto et al. genotyped 292 *P. vivax* isolates from Iquitos and 25 peri-urban and rural villages with 14 microsatellite markers. They identified Iquitos as a reservoir of parasite, spreading genetic diversity to the surrounding study areas (26). Similarly, Ventocilla et al. analyzed 145 *P. vivax* isolates from the Peruvian North Coast (PNC) using five microsatellite markers. They found low genetic diversity in the PNC but high diversity in the neighboring Ecuadorian Amazon Basin, indicating limited population flow between these regions separated by the Andes Mountains (27). Lastly, Manrique *et al*. conducted molecular surveillance in the Peruvian Amazon using 16 microsatellite markers on 777 mono-infection isolates. Their findings indicated gene flow of *P. vivax*, supporting the hypothesis that human mobility can potentially connect even geographically distant areas (12).

Among the studies conducted in Colombia, the earliest was by Osorio *et al*. in 2007. They used five microsatellite to classify 679 *P. falciparum* isolates into imported and indigenous. They found that microsatellite markers had low power to distinguish between these types due to a predominance of a single genotype and low genetic diversity in both imported and indigenous cases (19). Chenet *et al*. investigated the genetic composition of 257 *P. falciparum* isolates collected between 2002 and 2009. The findings suggested that fluctuations in the number of cases during this period were likely driven due to local rather than imported cases (28). Expanding the scope to inter-regional human migration, Pacheco et al. (29) evaluated the genetic differentiation of 624 *P. vivax* isolates across four geographically separated areas using eight microsatellite markers. Contrary to expectations of clear geographic structure, the study revealed only moderate genetic differentiation. This suggested a corridor between the northwest and the south Pacific Coast of Colombia, where migration of infected individuals enabled the spread of the parasite (29).

The primary focus of studies published in Africa was on Central Africa. Patel et al. (30) investigated whether microsatellite markers could identify the origin of malaria outbreaks among United Nations soldiers returning from the Democratic Republic of the Congo (DRC) to Guatemala. Using eight microsatellite markers, they found that the parasites in the soldiers were genetically similar to those from the DRC, suggesting the outbreak was likely due to *P. falciparum* imported cases from the DRC (30). In 2019, Roh *et al*. focused on distinguishing between imported and locally acquired *P. falciparum* infections in Eswatini. They genotyped 582 isolates using 26 microsatellite markers and found nearly identical levels of genetic diversity in both imported and local infections, making it challenging to differentiate between the two types of infections (31). In comparison, Tessema *et al*. integrated travel history, mobile phone data, and parasite genetics to study malaria connectivity within Namibia and across the Angolan and Zambian borders. By genotyping 4,643 *P. falciparum* isolates using 26 markers, they provided strong evidence that most cases in northeastern Namibia were attributed to local transmission. This finding was consistent with estimates derived from mobile phone and travel history data (24). Atuh et al. (32) examined the genetic connectivity of *P. falciparum* in Northwestern and Southwestern Cameroon using nine microsatellite markers. Despite high human migration between the regions, the analysis of 232 isolates revealed only a small degree of genetic similarity, indicating limited gene flow between these areas (32). In contrast to previous studies, Lo *et al*. used 14 microsatellites to assess gene flow patterns of both *P. falciparum* (*n* = 226) and *P. vivax* (*n* = 205) isolates in Ethiopia. The findings revealed that human migrations may promote parasites gene flow between the northern and eastern regions of the country (33).

We identified three studies conducted in Asia, all carried out in China. Liu Yaobao et al. (34) analyzed 128 *P. vivax* isolates from Central China using seven microsatellite markers. Despite low endemicity, they found high genetic diversity, indicating gene flow among central provinces. However, the markers were not effective in distinguishing local from imported infections (34). Similarly, Lo et al. (15) examined the genetic diversity of *P. vivax* across 895 samples from the Myanmar-China border using 11 microsatellite markers. They also observed high genetic diversity, suggesting that human migration facilitated parasite gene flow both locally and across the border (15). In contrast, Liu Yaobao et al. (35) focused on identifying the geographic origin of imported *P. vivax* infections. They analyzed 602 cases traveled from 26 sub-Saharan African countries to China using 26 microsatellite markers. Genetically related infections were found in people who traveled to the same country at about the same time. The findings demonstrated the potential of these markers to trace the origin of infections on a global scale (35).

Only two studies were conducted in Oceania. To understand the migration patterns of *P. vivax* in Papua New Guinea (PNG), Fola *et al*. genotyped 219 *P. vivax* isolates using ten microsatellite markers. The study identified three major genetic populations, with gene flow patterns aligning with human migration routes. Notably, gene flow was higher among Mainland parasite than among Island populations (14). In 2024, Sekine *et al*. traced the origins of *P. vivax* among 69 cases from Aneityum Island in Vanuatu. The results of genotyping with five microsatellite markers showed that the parasites responsible for the outbreak in 2002 were imported. However, the exact source(s) could not be determined (36).

### 3.3 Single nucleotide polymorphism markers (SNPs)

We found eight studies that used SNP markers to assess the geographic origin of malaria infections, with two of these studies conducted in Asia. In Sri Lanka, Dewasurendra et al. (37) investigated *P. vivax* origins using a 40-SNP barcode on samples collected from 2005 to 2011. Nine samples clustered with the South American group, indicating these isolates were most probably imported cases. However, the lack of travel histories made such inferences remain unconfirmed (37). In contrast, Chang et al. (4) integrated data from a 101-SNP barcode with travel survey and mobile phone data to assess malaria transmission countrywide in Bangladesh. They could detect likely imported cases among 1,412 malaria-positive cases (4).

We identified a study conducted in Peru, South America by Kattenberg *et al*. They used a 33-SNP barcode to predict the origin of *P. vivax* infections in the Peruvian Amazon. The samples from a border community showed high similarity to Brazilian isolates, likely indicating the introduction of *P. vivax* from Brazil. Moreover, seven out of 230 samples (3%) were predicted to originate from Vietnam, Afghanistan, and Iran. However, this finding could not be fully confirmed as incorrect predictions might have arisen due to missing data in the SNP barcode (38). In Central America, only one study in Honduras explored the population structure of both *P. vivax* and *P. falciparum* isolates. The results showed that two out of three isolates with chloroquine-resistant parasites were imported cases from Africa. In contrast, SNP barcodes could not provide any information about the origin of infection for *P. vivax* (39).

Lastly, we found four studies conducted on a global scale. The first study, published by Preston *et al*. in 2014, included 711 *P. falciparum* isolates from 14 countries and developed a 23-SNP barcode. The SNP barcode determined the geographic origin of parasite isolates with 92% accuracy, as confirmed by available data regarding the origin of each sample (40). The same approach was applied by Ba *et al*. to compare *P. vivax* population in Mauritania, West Africa, with parasite populations in other countries. The results of a 38-SNP barcode revealed that the Mauritanian samples (*n* = 100) formed a distinct geographical group, with slightly closer relatedness to Ethiopia than to the samples from Southeast Asia (41). In comparison, Trimarsanto et al. (42) used a smaller number of SNPs (*n* = 33) to predict the infection origin of 799 *P. vivax* isolates collected from 21 countries. The findings confirmed the capacity of this SNP panel to predict infection's country of origin accurately (42). The most recent publication was conducted by Phelan *et al*. in 2023. They used the largest number of genetic markers (*n* = 274), incorporating a combination of drug resistance mutations, mitochondrial SNPs, and a set of established SNP markers. Analyzing data from 7,152 *P. falciparum* and 1,502 *P. vivax* samples revealed that genetic markers could predict the geographical origin of infections, with accuracy rates of 96.1% at the continental level and 94.6% at the regional level (22).

### 3.4 SNPs and microsatellite markers

Khaireh *et al*. genotyped five microsatellites and five SNPs associated with pyrimethamine resistance in 181 *P. falciparum* isolates collected over an 11-year period (1998, 1999, 2002, and 2009) from the low malaria transmission setting of Djibouti. The most notable finding was the decline in genetic diversity among parasite populations over the study period. This was possibly due to a decline in malaria transmission from neighboring countries, particularly Ethiopia (23).

### 3.5 Targeted sequencing

We identified eight studies focused on targeted genome sequencing. Cui *et al*. investigated the genetic diversity of the *P. vivax* apical membrane antigen-1 (*PvAMA-1*) gene, an important vaccine candidate for vivax malaria, in 152 imported cases from China and 73 isolates from Myanmar. They found high genetic diversity and positive selection in this gene among imported cases along the China–Myanmar border, implying that traveling may affect the population structure and genetic characteristics of malaria on the Chinese side of the China–Myanmar border (43). Xu et al. (44) studied nucleotide diversity in the polymorphic region of the *rif* gene which is involved in immune evasion. High genetic differentiation was found between 403 malaria cases imported from Ghana to China and South Asian populations, including, Thailand, Vietnam, Myanmar, and Cambodia. The findings indicated that the *P. falciparum* populations have a substantial continental genetic structure (44). Ruybal-Pesantez et al. (45) analyzed nine genetic variants in the DBL*α* region of *var* genes, which are involved in immune evasion, to assess parasite relatedness. Most of the 58 *P. falciparum* isolates in Ecuador were closely related, indicating local transmission rather than importation from other regions. However, the genetic similarity of some isolates to those in South America, particularly from Colombia (*n* = 5) and Peru (*n* = 1), suggested historical parasite importation (45). To investigate the contribution of parasite importation on local transmission in Kenya, Markwalter et al. (46) analyzed data from 2,521 *P. falciparum* isolates together with their travel histories. Deep amplicon sequencing of the circumsporozoite protein (*csp*) and apical membrane antigen 1 (*ama1*) genes led to the identification of 72 *csp* haplotypes and 88 ama1 haplotypes. The findings suggest that travelers’ contribution to overall transmission was very limited, making the likelihood of establishing new transmission chains by travelers very unlikely (46).

Unlike other studies, Hamedi et al. genotyped nine Single Tandem Repeat (STR) markers on the *pvmdr1* gene, which is associated with drug resistance, particularly in *P. vivax*. They could not find any notable genetic differentiation between 23 imported cases from Afghanistan or Pakistan and 73 autochthonous cases in Iran (47). In Brazil, Griffing et al. genotyped blood spots collected on filter paper from the 1980s and 1990s across three regions to investigate internal migration of *P. falciparum*. They analyzed 190 samples using 28 microsatellite markers on the *dhfr* and *dhps* genes, known to be associated with sulphadoxine/pyrimethamine (SP) resistance. Their findings suggested that internal migration of the parasite between the regions might lead to admixture of parasite lineages (48). In one of the most recent studies, Holzschuh et al. sequenced 187 *P. falciparum* samples from two regions in Ethiopia. Unlike other research, they used multiplex amplicon deep sequencing to analyze 35 loci, including those associated with drug resistance. The findings suggested that individuals with recent travel history might initiate local transmission of the malaria parasite within the community which then led to ongoing local transmission (49). Pierre-Louis et al. examined drug-resistance genotypes among 234 U.S. travel-related *P. falciparum* infections from 2018 to 2021. They found that *Pfcpmp* alone was not a reliable marker for determining geographic origin. However, a panel combining 61 SNPs in *Pfcpmp* with 69 SNPs in *Pfs47* improved classification accuracy to about 95% at the continent level, highlighting the complementary benefit of sequencing these markers together (50).

### 3.6 Whole genome sequencing data

Only seven studies used whole genome sequencing (WGS) data with different approaches: one focused on the mitochondrial genome of *P. falciparum* (51) and two on *P. vivax* (21, 52) while the others examined the whole genome of *P. falciparum* (53) and *P. vivax* (54–56). In 2019, Schmedes et al. sequenced the *Plasmodium* mitochondrial genome to trace the origin of imported *P. falciparum* cases in the U.S. from 2014 to 2017. Of the 265 cases, 13 samples were mapped to specific regions, like Philippines and Ghana. However, most samples could not be traced due to the presence of common mitochondrial haplotypes (51). Earlier, in 2014, Rodrigues et al. obtained similar results by analyzing 348 mitochondrial genomes from *P. vivax* parasites worldwide and sequences from 69 imported *P. vivax* infections diagnosed in the United States. Given the genetic similarities between mitochondrial lineages from Africa, South Asia, Central Asia, and the Middle East, mitochondrial haplotypes could not accurately assign the geographic origin of malaria cases (52). The same approach was applied by Li et al. to investigate the geographical origin of *P. vivax* cases in Hainan, China. The origin of seven out of 14 imported cases could be inferred which was consistent with recorded travel histories. However, the origin of other imported cases could not be traced, raising questions about the suitability of using mitochondrial genomes in this context (21).

In 2020, Buyon et al. explored patterns of recent common ancestry among 59 *P. vivax* samples from Panama collected between 2007 and 2009 and 2017–2019. The results revealed that four samples with travel history did not show recent ancestry connections with other Panamanian samples; instead, they clustered with samples from a previous study conducted in Colombia. Nevertheless, there was no indication of outcrossing between the potentially imported parasites and the local Panamanian parasite population (54). On a larger scale, Benavente et al. used WGS data to determine the geographical origin of 433 *P. vivax* isolates from 17 countries worldwide. The findings showed that a 71-SNP barcode had high predictive power, successfully identifying the geographic origin with 91.4% accuracy (55). In comparison, Kattenberg et al. developed two assays based on WGS data to predict the geographic origin of imported *P. vivax* infections at the country level and to detect small-scale population genetic variations within Vietnam. Using a 72-SNP barcode, 327 out of 402 samples (88.8%) were correctly mapped to expected origin, as determined by travel history or the sample collection site (56).

On a smaller geographic scale, Morgan et al. used the WGS data to investigate the importation of *P. falciparum* parasites from the high-transmission regions of mainland Tanzania (*n* = 36) to the low-transmission areas of Zanzibar (*n* = 21). The findings suggested that human travel to high-risk malaria regions is the most likely source of parasite importation into Zanzibar (53).

### 3.7 Analytical frameworks

The methods used to analyze genetic data can be broadly grouped into three main groups: those that assess genetic diversity, examine population structure, and determine relatedness among populations or individuals. The most common methods involved assessing genetic diversity and population structure. In assessing genetic diversity, expected heterozygosity (HE) was the most commonly used method applied in 18 studies, accounting for 44% of the total (Table 2). This metric was primarily used to indirectly measure the impact of imported cases on genetic variation within the local populations, specifically in the context of longitudinal studies. These studies measured HE over a defined period allowing the identification and analysis of trends in HE. The primary assumption in these studies was that, in the absence of imported infections, the HE would remain relatively stable over time. In contrast, the introduction of imported infection was expected to alter the HE values. As a result, these studies did not necessarily aim to test specific hypotheses comparing HE between different time points. Instead, they focused on comparing the summary statistics of HE to observe broader trends in genetic diversity. The Fixation Index (FST) was the second most frequently used method, appearing in 15 studies (37%). FST was used to measure the genetic differentiation among populations, in some cases, extrapolating the FST as measure of gene flow.

**Table 2:** O**v**erview of the analytical methods employed in eligible studies.

Model/metric	Genetic diversity	Population structure	Relatedness	Study	*n*	Frequency (%)
Expected heterozygosity (HE)	Yes	No	No	Osorio et al. (19), Khaireh et al. (23), Chenet et al. (28), Ventocilla et al. (27), Roh et al. (31), Liu Yaobao et al. (34), Griffing et al. (48), Liu Yaobao et al. (34), Pacheco et al. (29), Delgado-Ratto et al. (26), Manrique et al. (12), Hamedi et al. (47), Fola et al. (14), Tessema et al. (24), Ba et al. (41), Holzschuh et al. (49), Kattenberg et al. (38), Sekine et al. (36)	18	44
Admixture model	No	Yes	No	Chenet et al. (28), Ventocilla et al. (27), Dewasurendra (37), Liu Yaobao et al. (35), Lo et al. (15), Pacheco et al. (29), Delgado-Ratto et al. (26), Manrique et al. (12), Atuh et al. (32), Cui et al. (43), Hamedi et al. (47), Lo et al. (15), Fola et al. (14), Xu et al. (44), Holzschuh et al. (49), Sekine et al. (36)	16	39
Fixation Index (FST)	Yes	No	No	Khaireh et al. (23), Preston et al. (40), Griffing et al. (48), Liu Yaobao et al. (34), Lo et al. (15), Pacheco et al. (29), Morgan et al. (53), Atuh et al. (32), Lo et al. (33), Fola et al. (14), Li et al. (21), Ba et al. (41), Li et al. (21), Xu et al. (44), Kattenberg et al. (38)	15	37
Principal Component Analysis (PCA)	No	Yes	Yes	Morgan et al. (53), Benavente et al. (55), Dewasurendra (37), Liu Yaobao et al. (34), Pacheco et al. (29), Buyon et al. (54), Cui et al. (43), Fola et al. (14), Ba et al. (41), Phelan et al. (22), Holzschuh et al. (49), Kattenberg et al. (38), Sekine et al. (36)	13	32
Multiplicity of Infection (MOI)	Yes	No	No	Ventocilla et al. (27), Roh et al. (31), Morgan et al. (53), Liu Yaobao et al. (35), Delgado-Ratto et al. (26), Atuh et al. (32), Hamedi et al. (47), Lo et al. (33), Tessema et al. (24), Ba et al. (41), Holzschuh et al. (49)	11	27
Neighbour-joining tree	No	Yes	Yes	Liu Yaobao et al. (35), Liu Yaobao et al. (34), Patel et al. (30), Hamedi et al. (47), Lo et al. (33), Ba et al. (41), Benavente et al. (55)	7	17
Haplotype network analysis	No	Yes	Yes	Delgado-Ratto et al. (26), Manrique et al. (12), Cui et al. (43), Fola et al. (14), Xu et al. (44), Holzschuh et al. (49), Kattenberg et al. (38)	7	17
Within-host infection fixation index (FWS)	Yes	No	No	Roh et al. (31), Morgan et al. (53), Atuh et al. (32), Buyon et al. (54), Tessema et al. (24), Kattenberg et al. (38)	6	15
Analysis of molecular variance	No	Yes	No	Pacheco et al. (29), Delgado-Ratto et al. (26), Manrique et al. (12), Lo et al. (33), Li et al. (21)	5	12
Bottleneck analysis	Yes	No	No	Lo et al. (15), Delgado-Ratto et al. (26), Manrique et al. (12), Lo et al. (33), Fola et al. (14)	5	12
Migration analysis (gene flow)	No	Yes	No	Lo et al. (15), Delgado-Ratto et al. (26), Lo et al. (33), Fola et al. (14), Ventocilla et al. (27)	5	12
Discriminant Analysis of Principal Components (DAPC)	No	Yes	No	Valdivia et al. (39), Kattenberg et al. (57), Holzschuh et al. (49), Kattenberg et al. (38)	5	12
Median-joining haplotype network	No	Yes	Yes	Rodrigues et al. (52), Chenet et al. (28), Griffing et al. (48), Schmedes et al. (51), Li et al. (21)	5	12
Identity by state analysis	No	No	Yes	Liu Yaobao et al. (35), Atuh et al. (32), Buyon et al. (54), Tessema et al. (24)	4	10
Genetic Differentiation Index (GST)	Yes	No	No	Roh et al. (31), Liu Yaobao et al. (35), Fola et al. (14), Tessema et al. (24)	4	10
Unrooted neighbour-joining trees	No	Yes	Yes	Hamedi et al. (47), Fola et al. (14), Ruybal-Pesantez et al. (45), Sekine et al. (36)	4	10
Maximum likelihood Phylogenetic tree	No	Yes	Yes	Valdivia et al. (39), Schmedes et al. (52), Li et al. (35), Phelan et al. (22)	4	10
Tajima's D	Yes	No	No	Preston et al. (40), Morgan et al. (53), Xu et al. (44)	3	7
Jost's Differentiation (D)	Yes	No	No	Liu Yaobao et al. (35), Fola et al. (14), Tessema et al. (24)	3	7
Effective population size (Ne)	Yes	No	No	Chenet et al. (28), Roh et al. (31), Morgan et al. (53)	3	7
Naive Bayes classifier	No	Yes	No	Trimarsanto et al. (42), Kattenberg et al. (57), Pierre-Louis et al. (50)	3	7
(Connectivity) Network analysis	No	Yes	Yes	Markwalter et al. (46), Kattenberg et al. (57), Ruybal-Pesantez et al. (45)	3	7
Allelic richness (RS)	Yes	No	No	Liu et al. (35), Fola et al. (14)	2	5
Index of discriminatory power (D)	Yes	No	No	Khaireh et al. (23), Patel et al. (30)	2	5
Nei's genetic diversity	Yes	No	No	Lo et al. (33), Fola et al. (14)	2	5
Genetic mixing index	No	No	Yes	Chang et al. (4)	1	2
Mutation-Scaled Migration Rates	No	Yes	No	Ventocilla et al. (27)	1	2
Nearest neighbour distances	No	No	Yes	Osorio et al. (19)	1	2
Linear discriminant analysis	No	Yes	No	Preston et al. (40)	1	2
Random Forests	No	Yes	No	Benavente et al. (55)	1	2
Bayesian coalescent-based method	No	No	Yes	Chenet et al. (28)	1	2
Multidimensional scaling	No	Yes	Yes	Liu et al. (35)	1	2
Factorial correspondence analysis	No	Yes	No	Khaireh et al. (23)	1	2
Ripley's K function	No	Yes	Yes	Osorio et al. (19)	1	2
eBurst algorithm	No	Yes	Yes	Khaireh et al. (23)	1	2
Bayesian phylogenetic tree	No	Yes	Yes	Rodrigues et al. (52)	1	2
Tree depicts	No	Yes	Yes	Pacheco et al. (29)	1	2
Principal Coordinate Analysis (PCoA)	No	Yes	Yes	Delgado-Ratto et al. (26)	1	2
Malaria transmission modeling*	No	No	No	Markwalter et al. (46)	1	2

* To estimate the reproductive number (R) and effective reproductive number (Rt), inclusive and exclusive of travelers.

The Admixture model was used in 16 studies (39%) to analyze population structure, providing insights into the contribution of different populations to the genetic composition of individuals and revealing historical and ongoing parasite migration across geographical regions. Principal Component Analysis (PCA) was employed in 13 studies (32%) to detect population structure and substructure (Table 2). Both the Admixture model and PCA uncovered patterns and structure in genetic data to some extent. However, as exploratory methods without hypothesis testing, their results were fundamentally more subjective than objective.

We found only five studies (12%) that applied methods capable of directly estimating potential evidence of migration between populations. Delgado-Ratto et al. (26) assessed human migration patterns in and around Iquitos City using a Bayes approach based on the coalescence theory. The findings showed that such a model can estimate the magnitude and direction of parasite importation. Unfortunately, the study did not collect any information on travel patterns, making it almost impossible to confirm the findings (26). To explore gene flow between the Peruvian North Coast and the Ecuadorian Amazon Basin, Ventocilla *et al*. developed two migration models using a Bayesian approach with MIGRATE-N. The models showed the presence of three distinct subpopulations with some level of migration occurring between them. These findings were consistent with the results obtained from the structure analysis (27). In a similar approach, Lo et al. estimated the intensity and direction of gene flow using a Bayesian approach implemented in BayesAss program. The bidirectional migration was observed between the north and east Ethiopia, indicating that human migrations may facilitate the parasite gene flow (33). In 2017, Lo et al. applied a similar method, using a Bayesian approach to estimate the posterior probability distribution of the proportion of migrants from one population to another. The findings revealed a greater migration rate from Myanmar to China. By contrast, migration from China to Myanmar was relatively small (15). Unlike the other studies, Fola et al. (14) used divMigrate-online software to estimate migration patterns among different geographic regions of Papua New Guinea. Their findings showed more migration within the mainland than within the Island region and relatively limited migration between them (14). In addition, we identified two studies that developed a novel method to determine if an isolate is imported. Chang et al. compared the geographic distance between isolates with geographic distances estimated based on genetic data. They quantified the probability of a geographic distance given an SNP difference by applying Bayes’ rule (4). In contrast, Tessema *et al*. developed an approach that combined parasite genetic data and travel history data to estimate local and cross-border importation in Namibia. Infections from Namibia were more found to be genetically related to those from southern Angola and Zambia, compared to northern Angola. This indicated parasite mixing within the geographically connected Namibia-Angola-Zambia regional zone (24).

Interestingly, we identified four studies employing machine learning approaches to detect imported malaria. In 2022, Trimarsanto et al. developed a Naive Bayes classifier to predict the origin country of the infection. This likelihood-based classifier demonstrated a high capacity to predict the infection's country of origin with 95% accuracy. However, its application was limited to situations where the genetic reference panel of a given country was available and had an adequate sample size (42). The same approach was employed by Kattenberg et al. in 2022 to predict the country of origin for imported *P. vivax* cases, achieving a high prediction accuracy of 88.8% (56). In contrast, Pierre-Louis et al. trained this classifier using *P. falciparum* genomes of known geographic origin to classify travel-related cases at the continental level. The findings demonstrated a high accuracy of 95% (50). Lastly, Benavente et al. applied the Random Forests approach to first identify the SNPs with the highest predictive importance and then to classify the geographical origin of *P. vivax* malaria. This method achieved a high predictive accuracy of 91.4% in identifying the geographic origin of the infection (55).

Among the 41 studies, only one used mathematical modeling to estimate the impact of travelers on the persistence of malaria transmission. The study modeled the reproductive number (R) and effective reproductive number (Rt) both with and without considering travelers. The findings indicated that the case reproductive numbers were approximately 1 and remained unchanged when travelers were excluded from the transmission networks, suggesting that malaria transmission is primarily driven locally rather than by travel to other regions (46).

## 4 Discussion

Understanding the spatial spread of malaria parasites, particularly in low transmission settings where imported cases can undermine local interventions, is crucial for effective control and elimination efforts (20). This review synthesizes evidence on the routine use of parasite genetic data to map imported malaria, offering insights into methods and approaches for national control programs and elimination strategies.

This review highlights the diverse methods used to analyze parasite genetic data for mapping imported malaria cases, emphasizing the strengths and weaknesses of each approach. The findings indicated that molecular techniques such as microsatellite genotyping can offer valuable insights into parasite population structure and migration patterns. These markers have been widely used to study genetic diversity and population dynamics in various settings, including the Peruvian Amazon (26), Central Africa (30), Asia (35), and Papua New Guinea (14). The findings revealed patterns of gene flow and population differentiation, highlighting the role of human migration in shaping malaria transmission. However, these studies had limitations, including low discriminatory power in regions with low genetic diversity and challenges in discriminating imported cases from local cases in areas with closely related parasite clones (31).

Similarly, SNP markers have been used to identify the geographic origin of infections. Studies conducted in diverse settings such as Sri Lanka, Bangladesh, and Honduras have used SNP barcodes to track parasite movement and evaluate population structure. The findings of these studies demonstrated the reliability of SNP markers in classifying imported infections on both regional and global scales (22). It is worth mentioning that, like microsatellite markers, SNP markers also lack sufficient resolution to discriminate between imported and local parasites on a smaller geographical scale, such as within a country, thereby limiting their applicability (39).

Compared to microsatellite and SNP barcodes, whole genome sequencing (WGS) offers a comprehensive approach to understanding the genetic makeup of malaria parasites, providing insights into their origin, transmission dynamics, and evolution. Its ability to analyze the entire genome allows for a more detailed assessment of parasite diversity and population structure than other molecular techniques (57). WGS provides high-resolution data, enabling researchers to identify genetic variations at the nucleotide level, including single nucleotide polymorphisms (SNPs), insertions, and deletions. This level of detail is invaluable for tracking the spread of parasite populations, identifying transmission routes, and detecting emerging drug resistance (58). Moreover, WGS can reveal fine-scale population structure, helping to differentiate between closely related parasite strains and accurately trace their origins. Additionally, WGS data can be combined with epidemiological and clinical information to provide a comprehensive understanding of malaria transmission dynamics, identify the primary sources of malaria importation, and assess the impact of this importation on the genetic structure of local parasite populations. Such insights can subsequently be used to inform and optimize targeted intervention strategies (59).

While WGS is considered a promising approach, it presents challenges in accurately determining the geographic origin of imported parasites. One fundamental limitation is the presence of common haplotypes, which can reduce the predictive accuracy of WGS analysis. Additionally, the infrastructure required for WGS analysis, including specialized equipment and expertise, presents a significant barrier to widespread implementation. Moreover, the high cost associated with WGS makes it impractical for routine use in all regions affected by malaria. Addressing these challenges will require collaborative efforts and investment in infrastructure and research capabilities (60). It is important to acknowledge that, despite its many advantages, WGS should not be relied upon exclusively. A well-designed study framework that considers the specific study settings and populations is essential. This framework should ensure that samples are collected, transported, and analyzed efficiently to maintain their integrity. Furthermore, it should integrate genetic data with information from traveler surveys and epidemiological surveillance to provide a comprehensive understanding of malaria transmission dynamics and the impact of malaria importation on local populations (59).

It is also important to acknowledge that while research on *P. falciparum* has advanced significantly, benefiting from a wider range of standardized genetic markers, *P. vivax* remains less well-characterized (61). This gap in established methodologies and genetic markers for *P. vivax* creates challenges in accurately tracing its transmission dynamics and identifying imported cases. The disparity between research on *P. falciparum* and *P. vivax* underscores the need for future studies to focus on developing and standardizing molecular markers specifically for *P. vivax*. Addressing this gap will be essential not only for improving malaria control strategies in regions where *P. vivax* is prevalent but also for enhancing our understanding of the global burden of this species (62).

The findings revealed that current approaches for determining imported malaria cases have limitations in accurately tracing changes in parasite genetics over time. These methods often rely on cross-sectional data and summary statistics, which provide snapshots of parasite diversity at specific time points but may not fully capture the complexities of parasite transmission dynamic (63). To address this limitation, future research should prioritize longitudinal studies that track parasite transmission over time. By analyzing genetic data from multiple time points, researchers can gain a deeper understanding of how parasites spread and evolve in response to different human mobility patterns and changes in environmental conditions. In addition, longitudinal analysis will enable researchers to evaluate the effectiveness of malaria control measures and pinpoint transmission hotspots associated with imported parasites (64). Ultimately, the findings of such longitudinal studies will be crucial for developing targeted interventions that could lead to the elimination of malaria.

With the advancement of new sequencing technologies and their growing use, large amounts of genetic data can now be obtained in a relatively short period. This abundance of genetic information is valuable for more accurately determining the transmission dynamics of the malaria parasite. However, to make the most of this potential, it is essential to use appropriate analytical methods capable of extracting meaningful insights from the data. In this context, utilizing state-of-the-art sequencing technology alongside advanced statistical learning methods, such as machine learning, can hold promise for enhancing the analysis of parasite genetic data and deepening our understanding of malaria transmission dynamics. Machine learning algorithms can efficiently process large-scale genomic datasets to identify complex patterns associated with parasite migration and population structure (65). However, integrating machine learning methods into malaria research requires careful consideration of data quality, model interpretation, and computational resources. Future studies should focus on refining machine learning algorithms and validating their capabilities in diverse malaria-endemic settings to maximize their utility (66).

The findings of this review highlight the essential role of parasite genetic data in supporting malaria elimination strategies, particularly in regions approaching malaria elimination where imported cases may undermine the success of these programs. By integrating genetic data into surveillance systems, national malaria control programs (NMCPs) can more accurately differentiate between imported and locally acquired infections (61). This distinction is crucial for tailoring interventions, such as implementing targeted vector control measures or allocating resources to high-risk areas with frequent importation events. Additionally, analyzing genetic data on a consistent basis can provide NMCPs with a systematic approach to assess the effectiveness of current control measures and refine elimination strategies. These insights can also help adjust policies as needed (67). For instance, if the majority of imported cases originate from neighboring regions, prioritizing cross-border collaboration and coordinated interventions could significantly enhance malaria control efforts within shared transmission corridors. By offering actionable recommendations for detecting and managing imported cases, the genomic surveillance of malaria can serve as a valuable resource for strengthening the capacity of NMCPs to address the complex dynamics of elimination efforts (68). In this context, integration of genetic data with appropriate statistical/mathematical methods is a crucial step toward achieving malaria elimination.

## 5 Conclusion

Understanding the spatial dynamics of malaria transmission, particularly in low-transmission areas where imported cases can significantly impact local control efforts, is crucial for effective malaria elimination strategies. This review highlights various molecular methods, including microsatellite genotyping, SNP profiling, and whole-genome sequencing, that have been used to map imported malaria. While each approach has its strengths, they also face limitations, particularly in distinguishing between imported and locally transmitted cases in areas with low genetic diversity. Whole-genome sequencing offers the most detailed insights into parasite diversity and transmission dynamics, but its high cost and infrastructure requirements limit its widespread application. Combining molecular data with epidemiological and traveler survey information, along with advanced statistical methods such as machine learning, holds significant potential for enhancing the detection of imported malaria cases. Moreover, there is a critical need to develop mathematical models to better understand the broader dynamics of malaria transmission, particularly in relation to human mobility. These models can help design more effective interventions by simulating different scenarios of parasite transmission and control measures. Future research is needed to refine methodologies for both genomic surveillance of malaria and data analysis to more accurately detect imported cases and their role in the persistence of malaria transmission. Ultimately, integrating these approaches will strengthen malaria control programs and contribute to global efforts to eliminate malaria.
